# Exploring the association between indoor air pollution (IAP) and anaemia among pregnant women in India

**DOI:** 10.1371/journal.pone.0343845

**Published:** 2026-03-02

**Authors:** Jenica Barnwal, Dilwar Hussain, Rakesh Chandra

**Affiliations:** 1 School of Health Systems Studies, Tata Institute of Social Sciences, Mumbai, India; 2 Centre for the Study of Regional Development, Jawaharlal Nehru University, New Delhi, India; 3 School of Health Systems Studies, Tata Institute of Social Sciences, Mumbai, India; University of Gour Banga, INDIA

## Abstract

**Background:**

Anaemia during pregnancy in India persists despite policy attention and nutritional interventions. While its primary determinants are well-recognized, the association between indoor air pollution (IAP) and anaemia among pregnant women in India remains conspicuously under-explored.

**Methods:**

This study examines the relationship between exposure to IAP and anaemia among pregnant women, using the National Family Health Survey (NFHS-5, 2019–21), with an analytical sample of 25,579 respondents. Descriptive statistics, chi-square tests, and multivariable logistic regression were used to assess the association between IAP exposure and anaemia, while accounting for socio-demographic, health, and behavioural characteristics.

**Results:**

IAP was significantly associated with higher odds of anaemia (COR: 1.43, 95% CI: 1.36–1.51). Pregnant women who were younger, had lower levels of education, resided in rural areas, belonged to scheduled tribe communities, and poor wealth index household were more likely to be anaemic.

**Conclusion:**

These findings suggest the need to integrate household energy transitions and indoor air quality improvements into maternal anaemia reduction strategies.

## Background

Anaemia during pregnancy remains a critical contributor to maternal morbidity, particularly in low-resource settings where the physiological demands of gestation are often unmet. Clinically, the condition involves low haemoglobin concentration and reduced red blood cell (RBC) levels [1]. Globally, its burden is unequally distributed, affecting approximately 14% of pregnant women in high-income countries and up to 46% in low- and middle-income countries (LMICs) [2]. This disparity is mainly due to inadequate diet, weak health systems, and lower awareness of maternal nutrition [1]. If left unaddressed, anaemia initiates a cascade of clinical complications that threaten the survival of both the mother and the fetus [3]. The condition also increases susceptibility to life-threatening infections [2].

During pregnancy, a substantial increase in blood volume elevates iron requirements to sustain adequate oxygen transport to the developing fetus. Without sufficient iron intake or stores, women are at risk of iron deficiency anaemia [4]. Alongside nutritional factors, including insufficient consumption of iron, folate, and vitamin A [5,6], environmental exposures warrant increased scrutiny. Indoor air pollution (IAP) is essential in this milieu. In many LMICs, where biomass fuels are widely used for cooking, exposure to IAP may contribute to anaemia [7]. Pollutants released from biomass combustion can induce systemic inflammation, leading to “anaemia of inflammation” [8] and further burdening anaemia during pregnancy.

In LMICs like India, IAP remains a prevalent public health concern, as many households still rely on biomass fuels such as wood, cow dung, and crop residues for cooking and heating. Recent estimates suggest that about two-fifths of households rely on these sources for daily energy needs [9]. Household exposure to IAP is compounded by inadequate ventilation. Many Indian homes lack standard mechanisms such as exhaust fans, chimneys, or cross-ventilated kitchen spaces, which are important for dispersing harmful emissions [10]. The incomplete combustion of these materials releases harmful pollutants, including fine particulate matter (PM2.5), carbon monoxide, and polycyclic aromatic hydrocarbons [11].

Pregnant women represent an especially vulnerable cohort due to the physiological shifts of gestation, characterized by elevated iron requirements and altered blood composition [12–14]. Furthermore, in the Indian setting, cooking is perceived as a predominantly female responsibility, a social norm that typically persists throughout pregnancy [15]. This gendered division of domestic labour results in sustained maternal exposure to IAP during a critical window when both maternal and fetal health are highly sensitive. Chronic exposure during this period may jeopardize the health of future generations by increasing the risk of child wasting, stunting, and mortality [16,17].

While anaemia among pregnant women in India has been considerably studied and linked to various socio-demographic correlates, such as economic status [18,19], geographical region [20], and educational attainment [21,22], the contribution of IAP has received comparatively less empirical attention. The current research base remains fragmented and context-specific. Prior research from Ethiopia [23], as well as micro-level studies from Nagpur [24] and rural Tamil Nadu [25] indicate that exposure to biomass smoke is associated with a higher risk of anaemia during pregnancy. However, there is a dearth of evidence examining this relationship at the national level in India.

Using data from the National Family Health Survey (NFHS-5), this study examines the association between IAP and anaemia among pregnant women, while accounting for socio-demographic factors. Our study intends to delineate the environmental and socio-structural correlates of maternal anaemia in India. In view of the consequences of anaemia for maternal and neonatal outcomes, and its alignment with the targets of the 2030 Sustainable Development Agenda, the findings have relevance for informing public health policy and clinical discourse [26].

## Methods

### Data source

**Fig 1:**
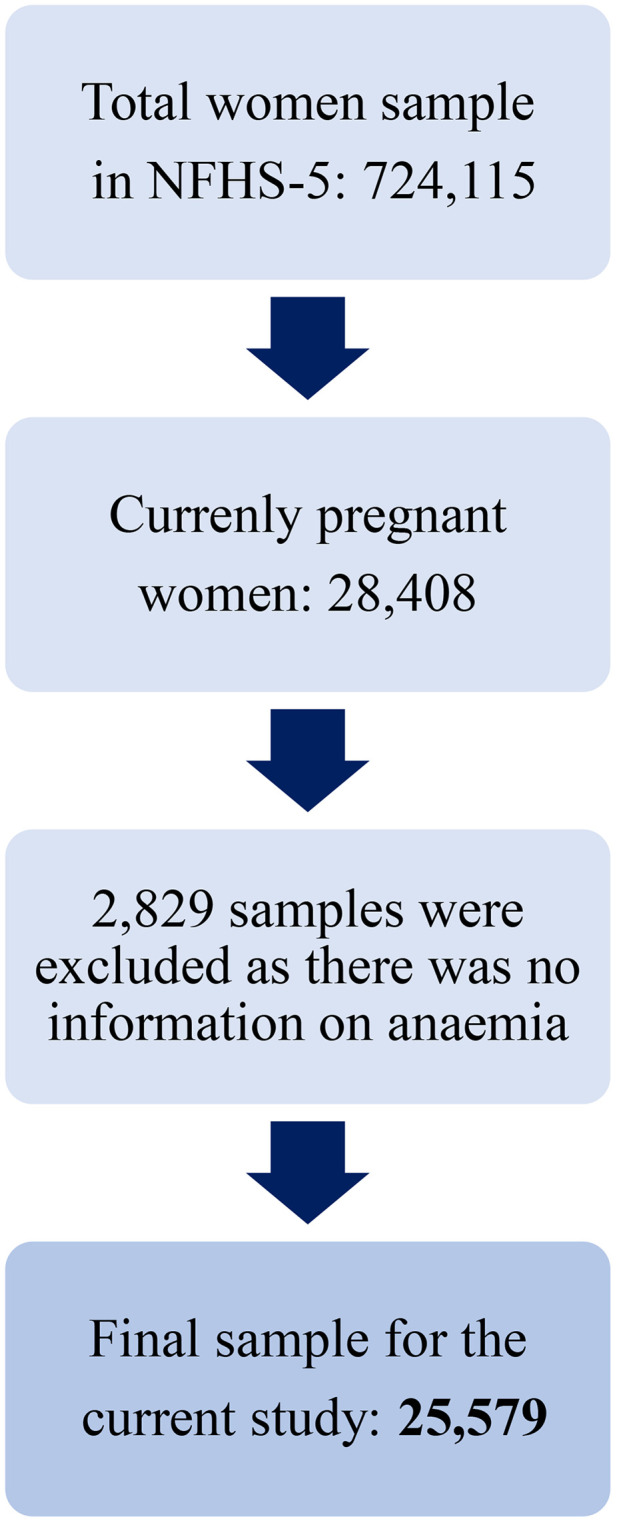
Sample selection procedure. A schematic flowchart representing the sample selection process from the NFHS-5 dataset. It shows the inclusion and exclusion criteria used to select the final analytical sample of pregnant women.

This study employed data from the National Family Health Survey (NFHS-5), conducted between 2019 and 2021. The NFHS-5 is a comprehensive, cross-sectional survey that collects data on various health indicators, including maternal and child health, family planning, reproductive health, and domestic violence. Detailed information on the sampling design and sample size is available in the national and state-level reports [27]. The survey included responses from 724,115 women aged 15–49 years, representing 636,699 households across 28 states and 8 Union Territories (UTs). For this analysis, a sample of 25,579 pregnant women from the same demographic and geographic scope was selected (see Fig. 1 for details on the sample selection process).

## Outcome variable

Anaemia was selected as an outcome variable for this study. It is categorized in the NFHS-5 dataset into four levels: no anaemia, mild anaemia, moderate anaemia, and severe anaemia, based on an ordinal scale. For pregnant women, mild anaemia was defined as haemoglobin levels between 10.0 and 10.9 g/dL. Moderate anaemia corresponded to a haemoglobin level of 7.0–9.9 g/dL. Severe anaemia was identified by haemoglobin levels below 7.0 g/dL [2]. To facilitate the analysis, the four categories were recoded into a binary variable: women with any level of anaemia (mild, moderate, or severe) were classified as “anaemic” (coded as “1”), while pregnant women with no anaemia were classified as “non-anaemic” (coded as “0”).

## Main predictor variable

The primary independent variable, IAP, was constructed using two key factors: the type of cooking fuel used and the location of cooking. The fuel type was divided into two groups: solid fuel (coded ‘1’, such as electricity, LPG, biogas, and kerosene) and clean fuel (coded ‘0’, such as coal, lignite, charcoal, wood, straw/shrubs/grass, agricultural crop residuals, and animal dung). The cooking location was recorded as a binary variable, with outdoor cooking coded as ‘0’ and indoor cooking coded as ‘1’. Households were considered to have IAP if they used solid fuel and cooked indoors. Women from such households were classified as exposed to IAP, with the variable categorised into two groups: not exposed (coded as ‘0’) and exposed (coded as ‘1’).

## Other predictor variables

Drawing from prior research on anaemia among pregnant women, the independent variables for this study were identified and categorised into four domains: (a) biodemographic and sociodemographic factors, (b) health-related variables, (c) behavioural variables, (d) exposure, and (e) wealth quintiles. The biodemographic and sociodemographic factors included age (grouped as 15–19, 20–29, 30–39, and 40–49), parity (0, 1–2, 3–5, and >5), place of residence (urban or rural), education level (categorized as illiterate, primary, secondary, or higher), and social category (SC, ST, OBC, and Others). Health-related variables included chronic conditions, such as the presence of asthma (yes or no). Behavioural variables encompassed alcohol consumption and smoking habits, both categorized as yes or no. Mass media exposure was assessed by determining access to newspapers, radio, and television at least once a week at the household level. This variable was classified into three categories: ‘No Exposure’, ‘Partial Exposure’, and ‘Full Exposure’. Wealth status was measured using the wealth index, which was based on household assets and utility usage and divided into five quintiles: poorest, poorer, middle, richer, and richest.

## Statistical analysis

The study analyzed the prevalence of anaemia among pregnant women in relation to IAP. Chi-square tests were used to assess the association between anaemia and predictors, and multivariable binary logistic regression was used to explore these relationships. Before conducting logistic regression, the variance inflation factor (VIF) was calculated to assess multicollinearity among the independent variables. Multicollinearity was not an issue for the models, as evidenced by the small VIFs (below 10) for each predictor variable. Four models were developed using block-wise forward selection to retain variables with p < 0.05. Model 1 focused on IAP exposure, Model 2 included biodemographic and socio-demographic factors (age, marital status, parity, education, social group, and residence), Model 3 added health-related and behavioral factors (BMI, asthma, smoking, and alcohol use), and Model 4 incorporated economic variables. Results were reported as adjusted odds ratios (AORs) with 95% confidence intervals (CIs) and p-values (<0.05). Analyses were conducted using STATA 17.

## Results

About 50.3% of the pregnant women were found to be anaemic, and nearly 46.5% were exposed to IAP (Table 1). Most of the women were aged 20–29 (71.2%), had one or two living children (49%), lived in rural areas (80.6%), were from OBC category (38.6%), and belonged to the poorest wealth quintile (24%). About 17% of pregnant women had no formal education, and a small proportion of pregnant women reported having asthma (0.8%), consuming alcohol (1.3%), or smoking (5.6%).

**Table 1:** Profile of the respondents, NFHS-5 (2019−21).

Variables	Frequency	Percentage (%)
*Outcome variable*		
**Anaemic**		
No	12,702	49.66
Yes	12,877	50.34
*Main predictor variable*		
**Exposed to Indoor Air Pollution**		
No	13,680	53.48
Yes	11,899	46.52
*Other predictor variables*		
**Age (in years)**		
15-19	2,617	10.23
20-29	18,228	71.26
30-39	4,495	17.57
43-49	239	0.93
**Parity**		
No child	10,520	41.13
1-2 children	12,555	49.08
3-5 children	2,335	9.13
>5 children	169	0.66
**Place of residence**		
Urban	4,963	19.4
Rural	20,616	80.6
**Educational level**		
Illiterate	4,319	16.88
Primary	2,817	11.01
Secondary	14,473	56.58
Higher	3,970	15.52
**Social category**		
SC	5,033	20.98
ST	5,716	23.82
OBC	9,264	38.61
Others	3,979	16.58
**Asthmatic**		
No	25,359	99.14
Yes	220	0.86
**Alcohol consumption**		
No	25,237	98.66
Yes	342	1.34
**Habit of smoking**		
No	24,133	94.35
Yes	1,446	5.65
**Wealth index**		
Poorest	6,299	24.63
Poorer	6,001	23.46
Middle	5,150	20.13
Richer	4,569	17.86
Richest	3,560	13.92

Table 2 shows that 57.3% of women exposed to indoor air pollution were anaemic, with higher prevalence among those without living children (53.5%), rural residents (57.6%), ST women (63.1%) and the poorest households (61%).

**Table 2:** Differentials in anaemia prevalence among pregnant women by background characteristics and IAP exposure, NFHS-5 (2019−21).

Variables	Anaemic (%)	P-value
**Exposed to Indoor Air Pollution**	57.32	
**Age (in years)**		0.001
15-19	56.99	
20-29	57.58	
30-39	56.29	
43-49	55.5	
**Parity**		<0.001
No child	53.56	
1-2 children	59.42	
3-5 children	60.82	
>5 children	61.4	
**Place of residence**		0.041
Urban	52.68	
Rural	57.65	
**Educational level**		<0.001
Illiterate	60.12	
Primary	59.42	
Secondary	57.09	
Higher	46.74	
**Social category**		0.008
SC	57.25	
ST	63.12	
OBC	54.47	
Others	59.33	
**Asthmatic**		0.926
No	57.33	
Yes	55.6	
**Alcohol consumption**		0.482
No	57.22	
Yes	70.91	
**Habit of smoking**		0.227
No	56.93	
Yes	66.39	
**Wealth index**		<0.001
Poorest	61.32	
Poorer	56.79	
Middle	51.37	
Richer	50.95	
Richest	54.01	

The findings from the logistic regression models are presented in Table 3. The unadjusted analysis showed that pregnant women exposed to IAP had a 43% higher likelihood of being anaemic (COR: 1.43, 95% CI: 1.36–1.51). In Model 2, after adjusting for socio-demographic factors, the association remained statistically significant, with IAP exposure associated with a 20% higher likelihood of anaemia (AOR: 1.20, 95% CI: 1.13–1.27). This association persisted after further adjustment for health-related and behavioural factors in Model 3 (AOR: 1.19, 95% CI: 1.12–1.26). In the fully adjusted model incorporating economic factors (Model 4), exposure to IAP remained significantly associated with anaemia (AOR: 1.07, 95% CI: 1.00–1.14). Anaemia was less likely among women aged 20−29 years (AOR: 0.76, 95% CI: 0.71–0.85), those with higher educational attainment (AOR:0.65, 95% CI: 0.59–0.72), and those in the wealthiest quintile (AOR: 0.65, 95% CI: 0.57–0.73). Moreover, asthma and smoking were not associated with anaemia.

**Table 3:** Logistic regression analysis assessing the impact of indoor air pollution on anaemia among pregnant women, NFHS-5, 2019−21.

Variables	Model 1 (Exposure to IAP)	Model 2 (Model 1 + Socio-Demographic factors)	Model 3 (Model 2 + Health-related and behavioural factors)	Model 4 (Model 3 + Economic factor)
*Main Predictor Factor*				
**Exposed to indoor air pollution**				
No ®				
Yes	1.43 (1.36, 1.51)***	1.20 (1.13,1.27)***	1.19 (1.12,1.26)***	1.07 (1.00,1.14)**
*Socio-demographic factors*				
**Age (in years)**				
15-19 ®				
20-29		0.76 (0.69,0.83)***	0.76 (0.71,0.85)***	0.79 (0.72,0.87)***
30-39		0.57 (0.51,0.65)***	0.58 (0.51, 0.65)***	0.61 (0.54, 0.69)***
40-49		0.50 (0.35,0.72)***	0.50 (0.35, 0.72)***	0.55(0.38, 0.79)**
**Parity**				
No child ®				
1-2 children		1.39 (1.31,1.48)***	1.39 (1.31,1.48)***	1.37 (1.29, 1.45)***
3-5 children		1.61 (1.44,1.81)***	1.61 (1.44,1.81)***	1.54 (1.37, 1.73)***
>5 children		1.81(1.26,2.61)**	1.81(1.28,2.67)**	1.70(1.17,2.45)**
**Place of residence**				
Urban ®				
Rural		1.15 (1.07, 1.23)***	1.14 (1.07, 1.22)***	1.05 (0.97, 1.12)
**Educational level**				
Illiterate ®				
Primary		0.94 (0.85,1.05)	0.94 (0.85,1.05)	0.97 (0.87,1.08)
Secondary		0.72 (0.81, 0.95)**	0.89 (0.82, 0.96)**	0.97 (0.89, 1.06)
Higher		0.65 (0.59, 0.72)***	0.67 (0.60, 0.74)***	0.79(0.71, 0.88)***
**Social category**				
SC ®				
ST		1.10 (1.00,1.21)**	1.08 (0.98, 1.20)	1.06 (0.98, 1.20)
OBC		0.86(0.79, 0.93)***	0.86 (0.79, 0.94)***	0.86(0.80,0.92)**
Others		0.83 (0.78, 0.89)**	0.83 (0.98, 1.20)	0.91(0.84, 1.00)***
*Health-related and behavioural factors*				
**Asthmatic**				
No ®				
Yes			1.20 (0.86, 1.66)	1.18 (0.85,1.63)
**Alcohol consumption**				
No ®				
Yes			1.24 (0.81,1.88)	1.20(0.79,1.82)
**Habit of smoking**				
No ®				
Yes			1.20 (1.01,1.42)**	1.16 (0.97,1.37)
*Economic factor*				
**Wealth index**				
Poorest ®				
Poorer				0.79(0.79, 0.93)***
Middle				0.68(0.68, 0.82)***
Richer				0.67(0.67, 0.82)***
Richest				0.52(0.52, 0.66)***

Note: ®: Reference Category; *** p < 0.01, ** p < 0.05, *p < 0.10, confidence interval = (value).

## Discussion

Anaemia affects over 40% of pregnant women in LMICs [23,28] and carries grave implications for maternal and child outcomes [29]. Emerging evidence suggests that household environmental conditions, such as IAP, may contribute to this burden. This study is among the first to assess the association between IAP exposure and anaemia among pregnant women in India at the national level. We found that about 57% of pregnant women exposed to IAP were anaemic. Results from logistic regression analysis indicated that exposure to IAP increased the likelihood of anaemia by 43%.

Exposure to indoor air pollutants during pregnancy can have long-lasting effects on both maternal and fetal health. These pollutants can impair fetal growth, increase the risk of preterm birth, and harm placental development [30]. IAP is known to contain harmful substances, including carbon monoxide, ultrafine particles, and particulate matter (PM2.5 and PM10), which can trigger systemic inflammation and oxidative stress [31]. Our study found that pregnant women exposed to IAP had a higher odds of being anaemic as compared to those not exposed, independent of other factors. This finding concurs with previous studies [23,24]. While the underlying mechanisms remain underexplored in the literature, it is suggested that particulate matter from cooking fuels can induce oxidative stress in RBCs, altering their shape and flexibility. These changes can contribute to anaemia [24,32,33]. Extended exposure to low concentrations of pollutants from kerosene and biomass fuels can affect the body’s ability to produce RBCs [34,35] and interfere with heme synthesis, an essential component of haemoglobin. Many studies have also found that exposure to IAP reduces haemoglobin levels, thereby increasing the risk of anaemia [9,15,36,37].

Our results further indicated that younger women were at a higher risk of anaemia, likely due to inadequate dietary iron intake combined with the additional iron demands of menstruation, pregnancy, and lactation [29], as also observed in earlier research [18,38]. Moreover, pregnant women with lower levels of education were more likely to be anaemic, possibly due to less awareness about nutritional needs and self-care during pregnancy [39]. Higher parity was associated with increased anaemia risk, in line with previous research [40,41], as repeated pregnancies can deplete iron stores through haemodilution and childbirth-related blood loss. [42,43]. Additionally, women residing in rural areas were more likely to be anaemic than their urban counterparts, a pattern observed in both African [23,44] and Asian countries [40,45]. Poor nutritional status, inadequate access to healthcare, and inadequate dietary diversity may underlie this rural–urban gap [44].

Over the past few decades, the Indian government has implemented various programs to address anaemia among women, including the *National Nutritional Anaemia Prophylaxis Programme*, the *National Nutritional Anaemia Control Programme*, and the *National Iron Plus Initiative*, which provide iron-folic acid supplements and nutrition education [46–48]. However, factors like infections, lifestyle changes, inadequate implementation, and poor program coordination continue to hinder progress. Simultaneously, programs such as the *Pradhan Mantri Ujjwala Yojana* and the *National Programme on Improved Chulha* aim to reduce IAP by promoting the use of clean fuels. Nonetheless, the impact of the COVID-19 pandemic on rural incomes highlights the need to review LPG prices and subsidies to increase accessibility, ensuring they remain affordable for those in the lowest wealth quintile. It is essential to note that most of the households in India lack standard ventilation tools, such as exhaust systems, which are crucial for removing indoor pollutants, making this study particularly relevant. The use of improved biomass stoves fitted with chimneys can reduce indoor air pollution and may help lower the prevalence of anaemia. Given the increased vulnerability of pregnant women to both nutritional deficiencies and environmental exposures, understanding how IAP contributes to anaemia in this cohort is especially critical. A combination of clinical and community-based research is necessary to fully understand these relationships and obtain a greater grasp of this association.

### Strengths and limitations

This study stands out as one of the first national-level assessments to examine the relationship between anaemia and IAP among pregnant women. It uses nationally representative data, allowing the findings to be generalised across India. However, the study also has some limitations. The cross-sectional nature of the data limits the ability to establish causality between IAP exposure and anaemia. Moreover, IAP exposure was assessed using proxy indicators such as the type of cooking fuel and kitchen location, which may not accurately reflect actual exposure levels due to data limitations. Additionally, while the interaction between age and IAP exposure, or the effects of cumulative IAP exposure over time, could provide valuable information, such analyses require longitudinal or retrospective exposure data, which are currently unavailable in the NFHS dataset. However, this study provides evidence to inform effective policy interventions to reduce anaemia among pregnant women in a developing country like India, where a large proportion of households still rely on biomass fuels for cooking.

## Conclusion

The study revealed that about 57% of pregnant women who were exposed to IAP were anaemic. We also unpacked that women who were exposed to IAP were more likely to be anaemic. Maternal anaemia can impact child health, leading to low birth weight and early morbidity. Reducing IAP exposure among pregnant women can improve both maternal and child health outcomes, contributing directly to the achievement of Sustainable Development Goal 3. Policies should prioritize increasing access to clean cooking fuels and improving household ventilation, especially for young pregnant women, those belonging to ST communities, living in rural areas, or in the lowest wealth quintile.
